# ENPP1 processes protein ADP‐ribosylation *in vitro*

**DOI:** 10.1111/febs.13811

**Published:** 2016-08-05

**Authors:** Luca Palazzo, Casey M. Daniels, Joanne E. Nettleship, Nahid Rahman, Robert Lyle McPherson, Shao‐En Ong, Kazuki Kato, Osamu Nureki, Anthony K. L. Leung, Ivan Ahel

**Affiliations:** ^1^ Sir William Dunn School of PathologyUniversity of OxfordUK; ^2^ Department of Biochemistry and Molecular BiologyBloomberg School of Public HealthJohns Hopkins UniversityBaltimoreMDUSA; ^3^ OPPF‐UKThe Research Complex at HarwellRutherford Appleton LaboratoryHarwell OxfordUK; ^4^ Division of Structural BiologyHenry Wellcome Building for Genomic MedicineUniversity of OxfordUK; ^5^ Department of PharmacologyUniversity of WashingtonSeattleWAUSA; ^6^ Department of Biophysics and BiochemistryGraduate School of ScienceThe University of TokyoJapan; ^7^ Department of OncologyJohns Hopkins University School of MedicineBaltimoreMDUSA; ^8^ Present address: Laboratory of Systems BiologyNational Institute of Allergy and Infectious DiseasesNational Institutes of HealthBethesdaMD20892USA

**Keywords:** ENPP1, mass spectrometry, PAR, PARP1, phosphodiesterase, poly(ADP‐ribose), post‐translational modification, protein ADP‐ribosylation

## Abstract

ADP‐ribosylation is a conserved post‐translational protein modification that plays a role in all major cellular processes, particularly DNA repair, transcription, translation, stress response and cell death. Hence, dysregulation of ADP‐ribosylation is linked to the physiopathology of several human diseases including cancers, diabetes and neurodegenerative disorders. Protein ADP‐ribosylation can be reversed by the macrodomain‐containing proteins PARG, TARG1, MacroD1 and MacroD2, which hydrolyse the ester bond known to link proteins to ADP‐ribose as well as consecutive ADP‐ribose subunits; targeting this bond can thus result in the complete removal of the protein modification or the conversion of poly(ADP‐ribose) to mono(ADP‐ribose). Recently, proteins containing the NUDIX domain – namely human NUDT16 and bacterial RppH – have been shown to process *in vitro* protein ADP‐ribosylation through an alternative mechanism, converting it into protein‐conjugated ribose‐5′‐phosphate (R5P, also known as pR). Though this protein modification was recently identified in mammalian tissues, its physiological relevance and the mechanism of generating protein phosphoribosylation are currently unknown. Here, we identified ectonucleotide pyrophosphatase/phosphodiesterase 1 (ENPP1) as the first known mammalian enzyme lacking a NUDIX domain to generate pR from ADP‐ribose on modified proteins *in vitro*. Thus, our data show that at least two enzyme families – Nudix and ENPP/NPP – are able to metabolize protein‐conjugated ADP‐ribose to pR *in vitro*, suggesting that pR exists and may be conserved from bacteria to mammals. We also demonstrate the utility of ENPP1 for converting protein‐conjugated mono(ADP‐ribose) and poly(ADP‐ribose) into mass spectrometry‐friendly pR tags, thus facilitating the identification of ADP‐ribosylation sites.

**Abbreviations**
ADPr: ADP‐ribose
ARH: ADP‐ribosylhydrolase
ARTC: ADP‐ribosyltransferase cholera toxin‐like
ARTs: ADP‐ribosyltransferases
ECM: extracellular matrix
ENPP2‐1‐8xHis: ENPP2‐1 chimera containing C‐terminal 8xHistidine tag
ENPP2‐1‐T: ENPP2‐1 chimera purified by TARGET tag
ENPP: ectonucleotide pyrophosphatase/phosphodiesterase
MAR: mono(ADP‐ribose)
MARylation: mono(ADP‐ribosyl)ation
MS: mass spectrometry
NAD: nicotinamide adenine dinucleotide
NPP: nucleotide pyrophosphatase/phosphodiesterase
PARG: PAR glycohydrolase
PAR: poly(ADP‐ribose)
PARPs: poly(ADP‐ribose) polymerases
PARylation: poly(ADP‐ribosyl)ation
pR/phosphoribose/R5P: ribose‐5′‐phosphate
PRAMP: phosphoribosyl‐AMP
PTM: post‐translational modification
SVP: snake venom phosphodiesterase
TARG1: terminal ADPr protein glycohydrolase
TLC: thin layer chromatography

## Introduction

Protein ADP‐ribosylation is a conserved post‐translational modification (PTM) involved in the regulation of many cellular pathways in both eukaryotes and prokaryotes 1, 2, 3, 4. There are several enzyme classes of protein ADP‐ribosyltransferases (ARTs) that are all able to transfer an ADP‐ribose (ADPr) group from β‐nicotinamide adenine dinucleotide (β‐NAD^+^) onto a specific protein acceptor with release of nicotinamide 1, 2. Some of the described protein ART families are the bacterial dinitrogen reductase ADP‐ribosyltransferases (DraTs), poly(ADP‐ribose) polymerases (PARPs), ART cholera toxin‐like (ARTCs) and sirtuins 1, 4, 5, 6, 7, 8, 9, 10, 11. In eukaryotes, the ARTCs are mostly extracellular proteins and may control immune responses 6, 7, 8, 9. Sirtuins are primarily known as protein deacetylases, but some of them can ADP‐ribosylate protein targets 10, 11.

Among ART enzymes, PARPs are the most studied. In humans, 17 members have been identified 1. They are intracellular proteins involved in many cellular processes, such as DNA damage repair, transcription, cell cycle progression, unfolded protein response, trafficking, mitosis, cell death and RNA metabolism 1, 3, 4, 9, 12, 13, 14, 15, 16. The majority of human ARTs, such as all the ARTC family members, sirtuins and 11 out of the 17 human PARPs, are able to transfer only a single ADP‐ribose subunit (mono(ADP‐ribose), or MAR) to target proteins 1, 17, 18, most commonly on acidic residues, such as aspartic and glutamic acid, and arginine residues 1, 7, 9, 19, 20, 21. However, ADP‐ribosylation has also been described for other amino acids, such as serines, threonines, phosphoserines, cysteines, lysines and diphthamides (reviewed in Ref. 20). Several PARP family members (e.g. PARP1, PARP2 and tankyrases) are able to produce long poly(ADP‐ribose) (PAR) chains by adding further repeating ADPr units (up to 200 units in length) via unique O‐glycosidic ribose–ribose bonds 1, 15, 22, 23, 24.

**Figure 1:**
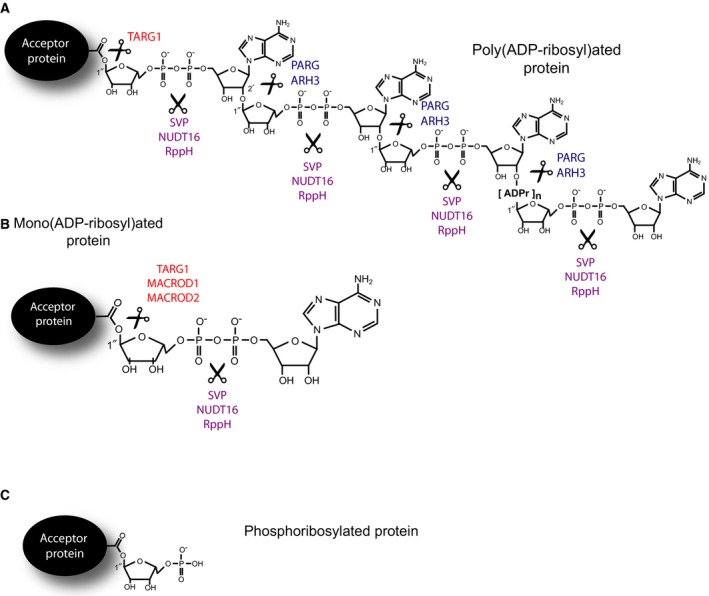
Metabolism of protein poly‐ and mono(ADP‐ribosyl)ation. Schematic illustration of protein PARylation (A) and MARylation (B). Enzymes and cleavable chemical bonds were indicated in figures. (C) Schematic illustration of protein phosphoribosylation.

Protein ADP‐ribosylation is a tightly controlled PTM 24, 25: once the cellular response induced by protein modification has been achieved, ADP‐ribosylation signalling has to be silenced properly and in a timely manner, and the ADPr subsequently recycled 1, 4, 26. PAR glycohydrolase (PARG) is the most characterized enzyme in humans for PAR hydrolysis, which specifically cleaves the ribose–ribose bonds between the ADPr subunits of the PAR chains (Fig. 1A) 27, 28. Another enzyme able to reverse protein poly(ADP)ribosylation (PARylation) is ADP‐ribosylhydrolase 3 (ARH3) 29. However, these two enzymes are unable to process MAR attached to a protein 28, 29. Glutamate‐linked MAR is known to be removed by macrodomain‐containing proteins such as terminal ADPr protein glycohydrolase (TARG1), MacroD1 and MacroD2 1, 30, 31, 32, 33 (Fig. 1B). Moreover, human ARH1 has been shown to remove MAR linked to arginine residues 34. In bacteria, mono‐ADP‐ribosylation (MARylation) mediated by DraT is reversed by DraG, a protein homologous to human ARH1/ARH3 proteins 5.

Recently, we showed that protein ADP‐ribosylation can undergo alternative processing 21, 26, 35: rather than complete removal of the ADPr group, the modification can be cleaved down to ribose‐5′‐phosphate (also known as phosphoribose/pR or R5P), a potentially toxic modification for which it is unclear whether and how it can be removed (Fig. 1C) 36. This reaction can be supported by phosphodiesterase I from *Crotalus adamanteus* (also referred to as snake venom phosphodiesterase, or SVP) 18, 37 as well as the nucleoside diphosphate‐linked moiety X (Nudix) family members human NUDT16 26 and RppH from *Escherichia coli* 35 (Fig. 1A–C).

Here, using PARP1 and PARP10 as *in vitro* models, we have identified a new enzyme, ectonucleotide pyrophosphatase/phosphodiesterase 1 (ENPP1), belonging to the family of nucleotide pyrophosphatase/phosphodiesterase (NPP) proteins 38, which is able to process protein ADP‐ribosylation to generate phosporibosylated proteins. This enzyme thus implicates a new class of proteins in the modulation of mammalian protein ADP‐ribosylation and the production of phosphoribosylated protein substrates. Such activity is consistent with the observation of phosphoribosylated proteins in a recent reanalysis of a phosphoproteome dataset 21. The identification of both acidic and basic protein ADP‐ribosylation sites has recently been made possible by the development of a mass spectrometry (MS)‐based method 19, 37, which relies upon the conversion of protein PAR and MAR into pR by the enzymes SVP 20, 37, NUDT16 26 or RppH 35. As a new member of this class of enzymes capable of converting protein‐conjugated ADP‐ribose to pR, we show here that ENPP1 can replace SVP for the identification of PARP1 and PARP10 automodification sites by MS.

## Results

### NPP‐type ectophosphodiesterases

**Figure 2:**
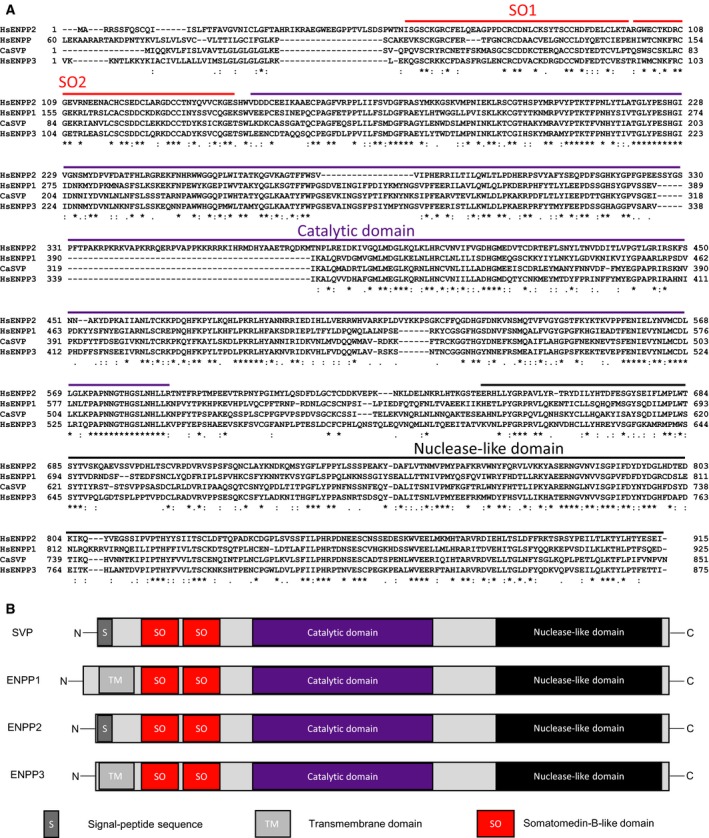
Alignment and domain analysis of ENPP proteins. (A) Clustal Omega alignment of amino acidic sequences belonging to SVP {gi|818935219|gb|JAI10403.1| phosphodiesterase [*Crotalus adamanteus*]}, human ENPP1 {gi|170650661|ref|NP_006199.2| ectonucleotide pyrophosphatase/phosphodiesterase family member 1 [*Homo sapiens*]}, human ENPP2, {>gi|91823274|ref|NP_006200.3| ectonucleotide pyrophosphatase/phosphodiesterase family member 2 isoform 1 preproprotein [*Homo sapiens*]} and human ENPP3 {>gi|111160296|ref|NP_005012.2| ectonucleotide pyrophosphatase/phosphodiesterase family member 3 [*Homo sapiens*]}. Legend to the alignment: ‘*’ positions which have a single, fully conserved residue; ‘**:**’ conservation between groups of strongly similar properties – scoring > 0.5 in the Gonnet PAM 250 matrix; ‘**.**’ Conservation between groups of weakly similar properties – scoring = < 0.5 in the Gonnet PAM 250 matrix. Domains and corresponding residues were highlighted. (B) Representative and schematic illustration of ENPP1, ENPP2, ENPP3 and SVP proteins. Legend of domains: S, signal peptide; TM, transmembrane domain; SO1, somatomedin B‐like domain 1; SO2, somatomedin B‐like domain 2.

Phosphoribosylation of cellular proteins has been detected recently 21. The human Nudix family member NUDT16 has been suggested as an enzyme that produces this modification by acting on ADP‐ribosylated proteins 26, 35. In the search for additional human enzymes able to process protein ADP‐ribosylation into pR, we looked for potential human homologues of snake venom phosphodiesterase I from *Crotalus adamanteus* (also known as SVP) 19, 20. SVP belongs to the class of highly conserved nucleotide pyrophosphatase/phosphodiesterase (NPP)‐type ectophosphodiesterases/extracellular glycoproteins 38. These enzymes are known to hydrolyse diesters of phosphoric acid into phosphomonoesters and can be classified, according to the nature of their substrate, into nucleotide and lipid phosphodiesterases 39. Currently, seven human genes are known to encode NPP protein homologues (ENPP1‐7). All human ENPP proteins are unrelated to phospholipases 40, Nudix hydrolases 41 or ectonucleotide triphosphate diphosphohydrolases 42. Among human NPP enzymes, only ENPP1, ENPP2 and ENPP3 share the same main domains of SVP in addition to the catalytic domain (Fig. 2A,B). Indeed, the mammalian ecto‐enzyme NPP2 (autotaxin) has a secretion motif in the N‐terminal domain, similar to SVP, while NPP1 (PC‐1) and NPP3 (B10; gp130RB13‐6) are characterized by a short N‐terminal intracellular domain and a single transmembrane domain (Fig. 2B). Except for these differences, all four enzymes share two somatomedin B‐like domains, a catalytic domain and a C‐terminal nuclease‐like domain. C‐terminal to the catalytic domain of NPP1–3 is the nuclease‐like domain, structurally similar to the DNA‐ or RNA‐nonspecific endonucleases 43. However, this domain is probably not catalytically active because none of the residues that are essential for catalysis by the nonspecific endoribonucleases is conserved in NPP1–3. Furthermore, the nuclease‐like domain is likely to harbour isoform‐specific determinants of catalysis because NPP2 with the nuclease‐like domain of NPP1 is inactive 44. Two somatomedin B‐like domains seem required for protein interaction similar to the somatomedin B domain of vitronectin 39. Members of the ENPP family hydrolyse various phosphodiester bonds (e.g. in oligonucleotides and artificial substrates like the p‐nitrophenyl ester of TMP) and pyrophosphate bonds (e.g. in (d)NTP, (d)NDP, NAD, FAD, diadenosine polyphosphates and UDP sugars) and thereby generate nucleoside 5′‐monophosphates. In particular, ENPP1 and ENPP3 show specificity to hydrolyse nucleotides 45. Thus, among the three mammalian ENPP enzymes, ENPP1 and ENPP3 display catalytic properties similar to SVP 46, 47. However, ENPP1 shows much higher hydrolytic activity than ENPP3 in the hydrolysis of various phosphodiester bonds 43.

**Figure 3:**
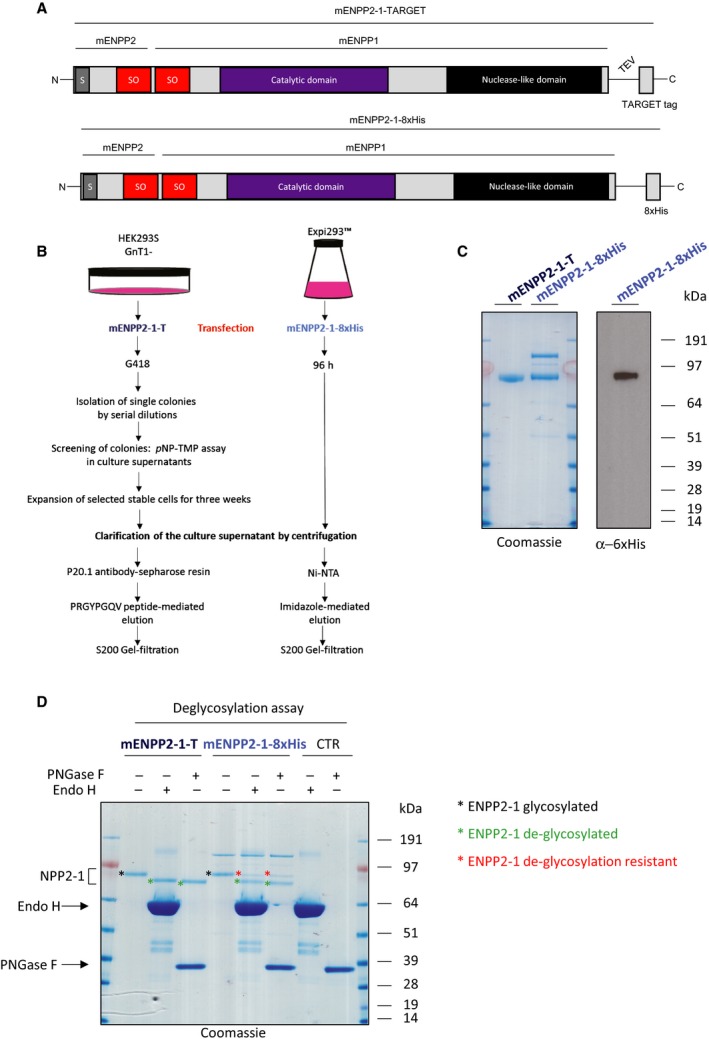
Production of ENPP recombinant proteins. (A) Representative and schematic illustrations of recombinant mouse ENPP2‐1‐Target (mENPP2‐1‐T) chimera purified as described in Kato *et al*. [42,43] and recombinant 8xHistidine tag version (mENPP2‐1‐8xHis). (B) Flow chart for the purification of mENPP2‐1‐T and mENPP2‐1‐8xHis. (C) Left panel, the purity of recombinant mENPP2‐1‐T and mENPP2‐1‐8xHis enzymes was analysed using separation of 20 μm of protein on an SDS/PAGE gel followed by staining with Coomassie. Right panel, 20 μm of mENPP2‐1‐T was probed by anti‐6xHis western blot. (D) 1 μg of mENPP2‐1‐T and mENPP2‐1‐8xHis enzymes were used as substrates for PNGase F and Endo H deglycosylation enzymes. Samples were resolved on SDS/PAGE and stained by Coomassie. Black star indicates glycosylated ENPP1, green star indicates deglycosylated ENPP1, red star indicates deglycosylation resistant ENPP1.

**Figure 4:**
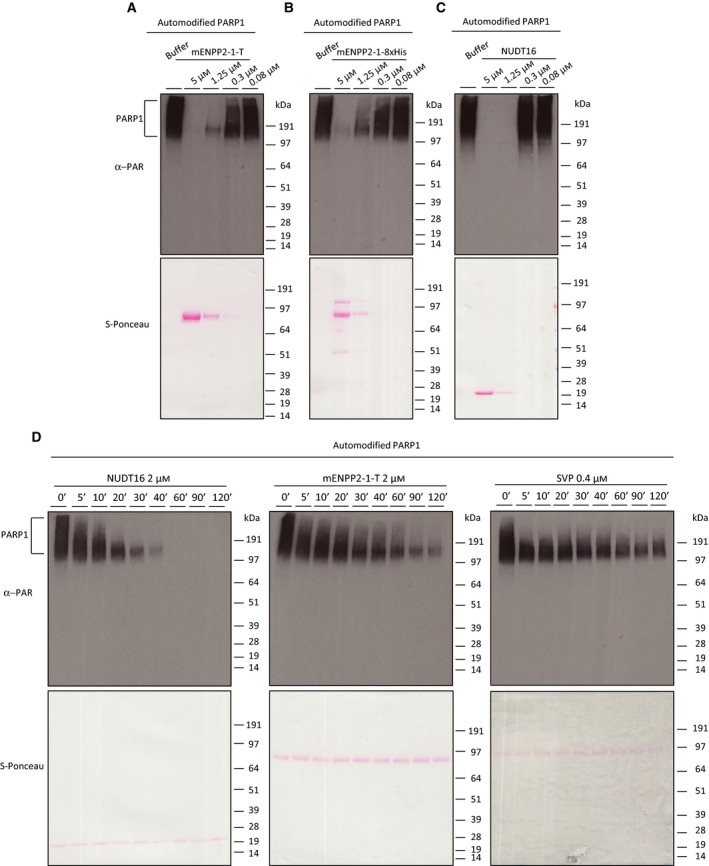
ENPP1 is able to hydrolyse protein poly(ADP‐ribosyl)ation. About 70 nm of human recombinant PARP1 was automodified to produce ~ 3 μm PAR substrate (defined in monomeric ADP‐ribose units) and incubated with buffer only (control) and decreasing concentrations of mENPP2‐1‐T (A), mENPP2‐1‐8xHis (B) and NUDT16 (C). Samples were fractionated on SDS/PAGE and transferred on nitrocellulose membranes. Membranes were first stained with S‐Ponceau and then probed with anti‐PAR antibody. (D) Time point hydrolysis of PARylated PARP1 was performed at indicated concentrations and times with NUDT16, mENPP2‐1‐T and SVP. Samples were resolved on SDS/PAGE and transferred on nitrocellulose membranes. Membranes were first stained with S‐Ponceau and then probed with anti‐PAR antibody.

Activity against ADPr and its use in proteomics of ADP‐ribosylated proteins has never been tested for mammalian ENPP proteins 19. In order to assay activity of mammalian ENPP proteins against protein ADP‐ribosylation, we focused on the well‐characterized mouse ENPP1 (mENPP1), which has 79% identity with the human ENPP1 protein 48, 49. To purify soluble ENPP1 enzyme, the extracellular and catalytic region of mouse Enpp1 was fused with the secretory signal sequence and the N‐terminal nine residues of the somatomedin B‐like 1 (SMB1) domain of mouse ENPP2 at the N terminus and, with the addition of a TARGET tag at the C terminus, we generated a recombinant, secreted ENPP2‐1 chimera amenable to enrichment via the TARGET tag system (mENPP2‐1‐T) (top panel Fig. 3A). The protein was then expressed in a clonal human cell line selected for higher activity against p‐nitrophenyl ester and purified using a specific antibody capturing the TARGET tag and isolating recombinant protein from cell media 48, 49 (left panel Fig. 3B). Of note, mENPP2‐1‐T was purified from HEK293S GnTI^−^ cells, cells depleted of N‐acetyl‐glucosaminyltransferase I (GnTI) activity and therefore lack complex N‐glycans 48, 49. We also produced an alternative construct for the expression and purification of ENPP2‐1 that would enable more accessible and easier purification. For this, we generated and purified an ENPP2‐1 chimera with a C terminus eight histidine tag (mENPP2‐1‐8xHis) from a pool of transiently transfected Expi293^™^ cells (Bottom panel Fig. 3A, right panel Fig. 3B). Comparison of mENPP2‐1‐T and mENPP2‐1‐8xHis protein preparations on SDS/PAGE (left panel Fig. 3C) showed a main band with a molecular weight of ~ 90 kDa. However, mENPP2‐1‐8xHis showed an additional high molecular weight contaminant protein. Anti‐6xHis western blot confirmed the presence of His‐tagged mENPP2‐1 protein at ~ 90 kDa (right panel Fig. 3C). To better investigate the glycosylation pattern in mENPP2‐1‐T and mENPP2‐1‐8xHis protein preparations, we performed deglycosylation assays treating both proteins with PNGase F (which removes almost all types of N‐linked glycosylation: high mannose, hybrid, bi‐, tri‐ and tetra‐antennary) and Endo H (which removes only high mannose and some hybrid types of N‐linked carbohydrates) (Fig. 3D). As expected, Coomassie staining revealed that mENPP2‐1‐T presents a homogeneous glycosylation pattern that can be reverted almost completely by PNGase F treatment (black stars, Fig. 3D). By contrast, mENPP2‐1‐8xHis showed a complex glycosylation pattern as neither PNGase F nor Endo H was able to completely compact the protein to a single band at a lower molecular weight (Fig. 3D). To test the activity of ENPP1 from both constructs against ADP‐ribosylated proteins, we incubated serial dilutions of purified mENPP1 proteins and NUDT16 – a positive control – with automodified PARP1 protein as a substrate for 3 h in the presence of 15 mm MgCl_2_. PAR was visualized by western blot using an antibody specifically recognizing poly‐ and oligo‐chains of ADPr but not MAR (Fig. 4A–C). Our data showed that ENPP1 successfully removes PAR chains from PARP1 protein with activity comparable to that exhibited by NUDT16, though mENPP2‐1‐T seems to be slightly more active than mENPP2‐1‐8xHis (Fig. 4A,B). Comparison of mENPP2‐1‐T and NUDT16 activity showed notably higher activity of NUDT16 against PARylated PARP1 (Fig. 4A,C). All the hydrolases exhibited time‐dependent activities (Fig. 4D).

### ENPP1 converts protein ADP‐ribosylation to protein phosphoribosylation

**Figure 5:**
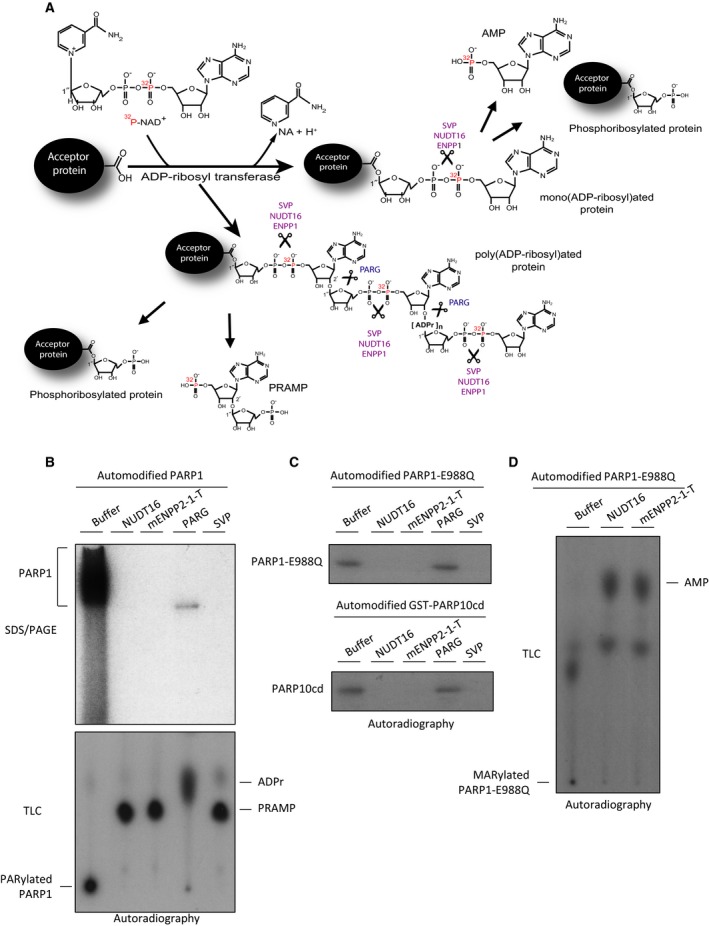
ENPP1 is able to hydrolyse protein poly‐ and mono‐(ADP‐ribosyl)ation producing PRAMP and AMP. (A) Schematic illustration of protein ADP‐ribosylation in the presence of NAD ^+^ labelled on the alpha phosphate group with ^32^P. Enzymes used in experiments showed in panels B–D, and cleavable chemical bonds in radiolabelled MAR/PAR were indicated. Main reaction products of phosphodiesterases‐dependent hydrolysis of radiolabelled protein PARylation were represented. (B) Human recombinant PARP1 was automodified in the presence of [^32^P]‐NAD ^+^ and then incubated with buffer (Control), 18 μm of recombinant NUDT16, 4 μm of recombinant ENPP2‐1‐T, 1 μm of PARG and 0.45 μm of purified SVP for 3 h at 30 °C. In top panel, samples were resolved on SDS/PAGE and [^32^P]‐NAD ^+^ incorporation was detected by autoradiography. In bottom panel, reactions described in top panel were loaded on TLC plate. (C) Top panel, 1 μm of recombinant PARP1‐E988Q mutant was automodified using ^32^P‐labelled NAD ^+^ and then incubated with buffer only (control), 5 μm of recombinant NUDT16, 5 μm of recombinant mENPP2‐1‐T or 2 μm of purified SVP. Samples were resolved on SDS/PAGE and [^32^P]‐NAD ^+^ incorporation was detected by autoradiography. Bottom panel, 1 μm of recombinant GST‐PARP10cd was automodified and treated as indicated in top panel. (D) The products of indicated enzymatic reactions were assayed by TLC.

Hydrolysis assays were again performed, this time using a more sensitive assay in which PARP1 is automodified in the presence of NAD^+^ labelled on the alpha phosphate group with ^32^P (Fig. 5A). PARylated PARP1 was then incubated with mENPP2‐1‐T, PARG (an enzyme known for removing PAR but leaving MAR) or the positive controls SVP and NUDT16 (Fig. 5A,B). As expected, ENPP1 was able to completely remove the radiolabelled PAR signal from PARP1, mirroring the phosphodiester hydrolysis activity of NUDT16 and SVP and failing to leave MAR at the attachment site, as PARG does (Fig. 5A,B). The main product of this reaction was phosphoribosyl AMP (PRAMP) as seen for SVP and NUDT16 previously 19, 26, 37 (Fig. 5A), [thin layer chromatography (TLC)] (bottom panel Fig. 5B).

In order to directly address whether ENPP1 is able to remove MAR from proteins, recombinant PARP1–E988Q, a PARP1 mutant able to add only a single unit of ADPr onto target proteins (in this case, itself), and a mono‐ADP‐ribosyl transferase GST‐PARP10 catalytic domain, were automodified and then incubated with NUDT16, mENPP2‐1‐T, PARG or SVP (Fig. 5C). mENPP2‐1‐T removed the ^32^P‐labelled MAR signal as efficiently as SVP and NUDT16, thus proving that mENPP2‐1‐T is active against protein‐conjugated MAR. The TLC analysis showed that, as expected, the main reaction product is AMP (Fig. 5D). Taken together, we conclude that ENPP removes PARP‐dependent protein ADP‐ribosylation.

### ENPP1 efficiently removes ARTC2.2‐mediated ADP‐ribosylation

**Figure 6:**
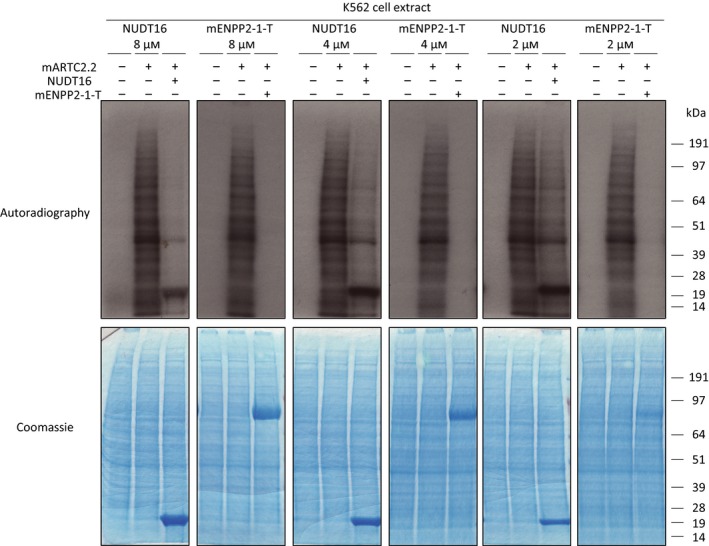
ENPP1 efficiently *in vitro* neutralize ARTC2.2‐dependent ADP‐ribosylation of cellular proteins. (A) K562 cell extract was supplemented with 37 kBq of [^32^P]‐labelled NAD ^+^ and 15 mm MgCl_2_. Then, the extract was incubated with or without 1 μm of recombinant mARTC2.2 for 15 min. Subsequently, the extract was incubated with or without NUDT16 or mENPP2‐1‐T at the indicated concentrations. Top panel, samples were resolved on SDS/PAGE and [^32^P]‐NAD ^+^ incorporation was detected by autoradiography. Bottom panel, Coomassie staining of dried gel exposed in top panel.

As mentioned before, ENPP1 is an ecto‐enzyme initially described as plasma cell membrane glycoprotein 1 (PC‐1, CD203); however, its involvement in lymphocyte biology has not been studied thoroughly 30, 38, 44, 49. Localization of ENPP1 to the cell surface suggests that ENPP1 may process ADP‐ribosylation synthesised by extracellular ADP‐ribosyl transferases such as membrane‐bound cholera‐like ecto‐ADP‐ribosyltransferases (ARTCs) 7, 8, 9. Unlike PARPs, these enzymes usually modify protein arginine residues 6, 7. In particular, we focused on the ARTC2.2 protein expressed as an ecto‐enzyme on the plasma membrane of mouse immune cells 50. ARTC2.2 is known to have a crucial regulatory function on the activity and survival of T lymphocytes and NK lymphocytes, ADP‐ribosylating several proteins, such as the purinergic P2X7 receptor, LFA‐1 and CD8 50. To address the question of whether ENPP1 can remove ARTC2.2‐dependent ADP‐ribosylation in a cellular context, we expressed and purified the catalytic domain of mouse ARTC2.2 (mARTC2.2) in and from *E. coli* and then *in vitro* incubated the ART recombinant protein with K562 (human NK lymphocyte) cell extract in the presence of radiolabelled NAD^+^ (Fig. 6A). Under these conditions, mARTC2.2 was able to reproducibly modify several proteins in the K562 cell extract. Concomitant incubation of mARTC2.2 modified extract with NUDT16 showed significant reduction in radiolabelled ADP‐ribosylated proteins; however, incubation of cellular proteins with the same concentrations of mENPP2‐1‐T gave rise to an even more remarkable hydrolysis of mono‐ADP‐ribosylated proteins. Of note, NUDT16 was itself modified by mARTC2.2. Our data show that ENPP1 can better perform conversion of ADP‐ribosylated proteins into pR‐proteins than NUDT16 in this cell‐free system and reaction conditions. Taken together, our data demonstrate that ENPP1 is able to remove ARTC‐mediated ADP‐ribosylation *in vitro*, and suggest that this event may occur *in vivo* due to the extracellular proximity of ARTCs and ENPPs.

### ENPP1 as a tool for LC‐MS/MS aided identification of protein ADP‐ribosylation sites

**Figure 7:**
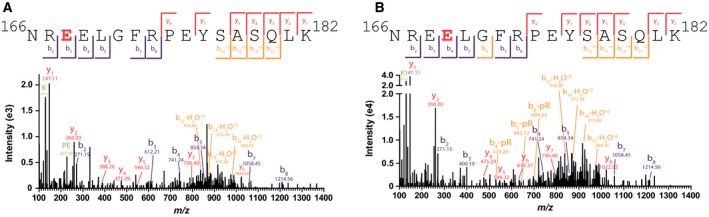
ENPP1 hydrolyses poly(ADP‐ribose) to pR, a molecular tag detectable by LC‐MS/MS. (A) PARP1 carries a 212.01 Da shift representative of pR on E168. (B) PARP1 carries pR on E169, as shown here clearly distinguishable from the pR‐E168 peptide form. Both peptides detected following digestion of PAR by ENPP2‐1‐T in a 10× enzyme:PARP ratio.

In order to determine whether ENPP2‐1 can serve as a replacement for SVP in the recently established ADPr site identification proteomics pipeline 19, 35, we treated 60 pmoles of autoPARylated PARP1 protein with 120 pmoles of SVP, mENPP2‐1‐T or mENPP2‐1‐8xHis, or 600 pmoles of mENPP2‐1‐T or mENPP2‐1‐8xHis and used LC‐MS/MS to search for the 212.01 Da shift characteristic of pR‐modified peptides. Ten PARP1 peptides confidently presented with an MS1 mass shift corresponding to a singly or doubly phosphoribosylated state combined with peptide sequencing by MS2 to determine site localization (Table 1). As shown in Fig. 7, panels A and B, the ambiguity in site localization as reported by MaxQuant can often be overcome by *de novo* sequencing of the spectrum of interest, shown here for the first two forms of peptide 3, where pR is carried on E168 (panel A) and E169 (panel B). A direct comparison between the peptide forms identified following SVP, mENPP‐2‐1‐T or mENPP‐2‐1‐8xHis digestion is depicted in the ‘2×’ columns of Table 1, wherein the enzymes were incubated with PARylated PARP1 in a 2 : 1 molar ratio (note that this ratio is for enzyme:protein, not enzyme:substrate, where the substrate would be ADPr). Of the 19 peptide forms identified in this analysis, 15 were found following exposure to SVP, 16 following mENPP‐2‐1‐T and 11 following mENPP‐2‐1‐8xHis, demonstrating the comparability of these three enzymes in this application. To determine whether these reactions can be driven to completion by the addition of excess enzyme, the ENPP1 proteins were added in a 10 : 1 enzyme/PARylated protein ratio (see Table 1, columns labelled 10×), resulting in 19/19 peptide forms being identified in the mENPP‐2‐1‐T sample and 9/19 peptide forms showing up in the mENPP‐2‐1‐8xHis sample. This result suggests that, at least in the case of the highly active form of ENPP1 (mENPP‐2‐1‐T), the transformation of PAR to pR can be driven to completion by the addition of more recombinant enzyme.

**Table 1:**
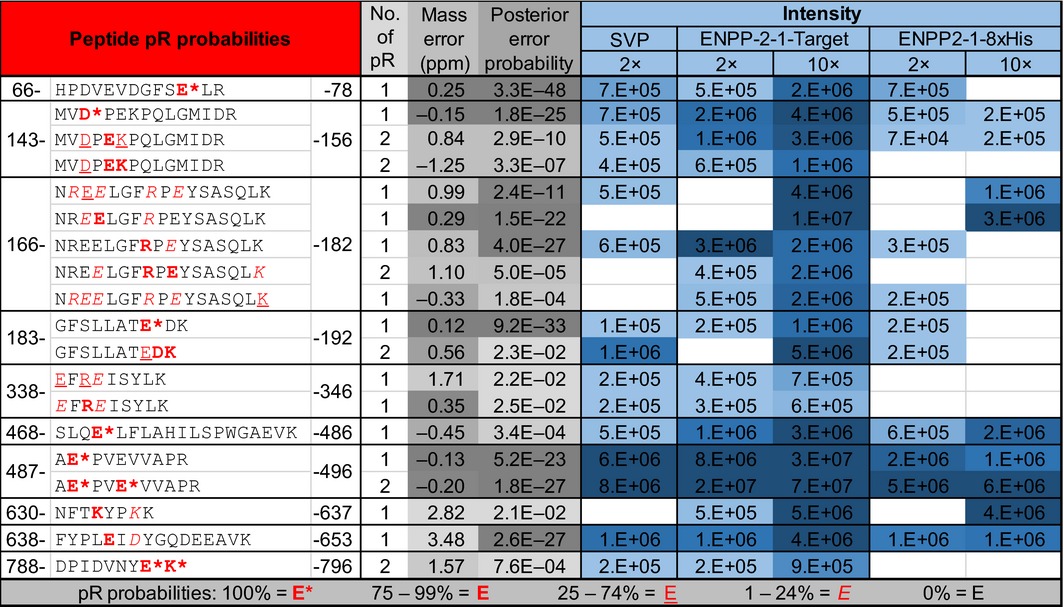
ENPP1 as a tool for LC‐MS/MS aided identification of protein ADP‐ribosylation sites. PARP1 automodification sites identified using the pipeline described by Daniels *et al*. 19. Experimental conditions were described in the main text. Posterior error probabilities (PEPs) are reported for corresponding amino acid residues as well as site localization probabilities within the identified pR‐containing peptides. The 2× and 10× columns refer to the reaction ratio of enzyme to PARylated PARP1. Key at the bottom describes the confidence ranges reported by MaxQuant for site identification (with possible modification sites of D, E, K and R); often this ambiguity can be resolved by manual inspection of the peptide fragmentation pattern (see Fig. 7 for annotation of two different forms of peptide N166‐K182)

In order to confirm that ENPP1 is able to convert protein‐conjugated mono(ADP‐ribose) to a pR tag, we automodified the catalytic domain of PARP10 – an enzyme restricted to mono(ADP‐ribosyl)ation activity – and exposed it to mENPP‐2‐1‐T. This resulted in the confident identification of four pR‐containing PARP10 peptides after ENPP1 treatment (Table 2). K916, a residue previously identified as a PARP10 automodification site 18 and also known to be acetylated 51, was among the identified mono(ADP‐ribosyl)ated sites.

**Table 2:**
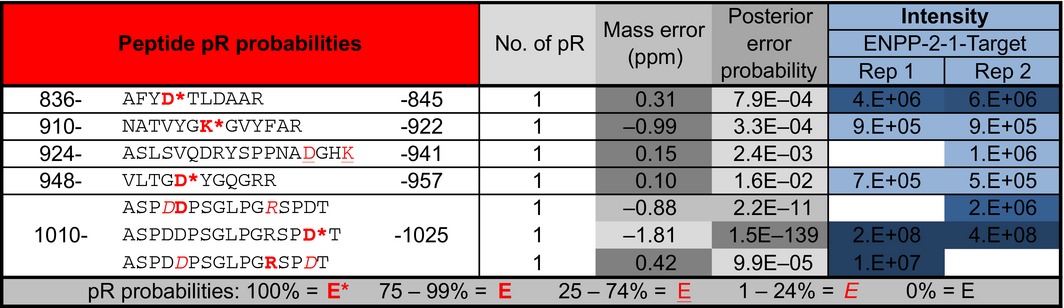
ENPP1 as a tool for LC‐MS/MS aided identification of protein mono‐ADP‐ribosylation sites in PARP10 catalytic domain. Posterior error probabilities (PEPs) are reported for corresponding amino acid residues as well as site localization probabilities within the identified pR‐containing peptides. Two replicates for mENPP‐2‐1‐T treated samples are shown. Key at the bottom describes the confidence ranges reported by MaxQuant for site identification

## Discussion

ENPP1 is a type II transmembrane glycoprotein with nucleotide phosphodiesterase activity 38, 39. This protein has broad specificity and cleaves a variety of substrates, including phosphodiester bonds of nucleotides and nucleotide sugars, and pyrophosphate bonds of nucleotides and nucleotide sugars. This protein may function to hydrolyse nucleoside 5′ triphosphates to their corresponding monophosphates and may also hydrolyse diadenosine polyphosphates 38, 39, 46, 47.

Mutations in the ENPP1 gene have been associated with ‘idiopathic’ infantile arterial calcification 52, ossification of the posterior longitudinal ligament of the spine OPLL 53, hearing loss 54 and insulin resistance 49. ENPP1 has been described as essential for physiological mineralization because its expression on the outer surfaces of mineralizing cells, such as osteoblasts and chondrocytes, regulates the balance between the extracellular concentrations of inorganic phosphate (Pi), a substrate for mineralization, and inorganic pyrophosphate (PPi), an inhibitor of mineralization 55. Although ENPP1 is essential for the regulation of physiological mineralization, its substrate specificity for different nucleotides is not completely described.

While aligning the nucleotide sequences of the ectonucleotide pyrophosphatase/phosphodiesterase enzymes with SVP, we hypothesized that ENPP1 could act in a similar manner as SVP to convert ADP‐ribosylated proteins into proteins containing a pR tag. In addition to the wide range of intracellular roles played by PAR, its recently observed presence in the extracellular matrix (ECM) suggests an extracellular function for this polymer 56. In particular, PAR was identified in the ECM of developing bones, where it was likely released from necrotic osteoblasts and playing an essential role as a scaffold for bio‐mineralization 56.

Our biochemical data suggest that ENPP1 may be involved in the metabolism of extracellular PAR through its hydrolysis and release of phosphoribosylated proteins, PRAMP and, contextually, free PAR – a molecule that has previously been described as an extracellular stimulus driving inflammatory signalling 57. Moreover, colocalization of ARTCs and ENPP1 on the extracellular membrane 50 may suggest a physiological function for ENPP1 in the regulation of immune cell activity and survival.

The advent of mass spectrometry‐based methods for identifying ADP‐ribosylation sites has allowed for large‐scale assessments of the endogenously ADP‐ribosylated proteome at the protein sequence level in human and murine cells 19, 58. All of the available methods for PAR site identification rely upon the creation of a molecular ‘tag’ at the PTM's amino acid attachment site 20, in the case of SVP digestion this tag is pR. SVP, however, must be purified from snake venom through a multi‐step process, resulting in a high prep–to–prep variation due to both source variability (at the level of the snake venom) and inevitable inconsistencies between protein purification pipelines (at the level of instrumentation, reagents, etc.) 26, 35. Therefore, in order to broadly implement the pR pipeline for proteomics applications, it is necessary to replace SVP with a phosphodiesterase that can be made in large quantities and with high consistency (as a recombinant protein, expressed and purified in the laboratory). We have presented two options for this purpose: mENPP2‐1‐T and mENPP2‐1‐8xHis. Of these two, the former (mENPP2‐1‐T) has proven to be a more active form of this enzyme, while the latter (mENPP2‐1‐8xHis) can more easily be adapted for expression and purification in standard laboratories equipped to purify His‐tagged proteins. The availability of these tools will allow for greater adoption and implementation of the pR‐based proteomics pipeline for the unbiased identification of protein mono and poly(ADP‐ribosyl)ation sites.

## Materials and methods

### Plasmids and recombinant proteins

PARP1 wild‐type protein was purchased from Trevigen Inc., (Gaithersburg, MD, USA) (high specific activity) for biochemical assays or expressed and purified as previously described 19 for analysis by mass spectrometry. PARP1‐E988Q was expressed from pET28a(+) and purified as previously described 30. NUDT16 was expressed from pNIC28‐Bsa4 and purified as described in Palazzo *et al*. 26. pGEX‐4T1 GST‐PARP10cd (amino acids 818–1025) plasmid was a gift from Bernhard Lüscher (RWTH Aachen University) 17. GST‐PARP10cd recombinant protein was purified from transformed Rosetta2 (DE) competent cells. Briefly, transformed bacteria were grown over night in LB supplemented with 100 μg·mL^−1^ of ampicillin and 34 μg·mL^−1^ chloramphenicol at 37 °C. Overnight culture was diluted in 4 L of media and grown at 37 °C until the absorbance measured at 600 nm reached 0.8. Temperature was then cooled down to 18 °C, bacteria were induced with 0.2 mm IPTG and culture was prolonged for 16 h. Bacteria were then lysed using BugBuster protein extraction reagent [Novagen (Merck Biosciences), Beeston, Nottingham, UK] and Benzonase (Sigma‐Aldrich Company Ltd., Dorset, UK) in PBS buffer supplemented with 10% glycerol, 1 mm DTT and Complete Protease Inhibitor [Roche Products Limited (Pharmaceuticals), Welwyn Garden City, UK]. After 1‐h incubation, the lysate was clarified by centrifugation and supernatant applied on glutathione sepharose beads (GE Healthcare, Amersham, UK). Beads were incubated with lysate for 50 min at 4 °C and then washed in 20 column volumes of lysis buffer. GST‐tagged protein was eluted in lysis buffer supplemented with 20 mm reduced glutathione (Sigma, readjusted pH to 7.4). Fractions were then collected, assayed by SDS/PAGE and Coomassie blue staining (Instant Blue; Expedeon LTD, Swavesey, UK). Best fractions were pulled and dialysed in 25 mm Tris/HCl pH 7.5, 150 mm NaCl, 10% glycerol, 1 mm DTT. mENPP2‐1‐T was expressed from pcD‐CW vector as described in detail in Kato *et al*. 48, 49. In particular, the extracellular region of mouse Enpp1 (residues 92–905) was fused with the secretory signal sequence (residues 1–50) and the N‐terminal nine residues of the somatomedin B‐like 1 (SMB1) domain (residues 51–59) of mouse Enpp2 at the N terminus and with the TARGET tag at the C terminus to generate a secreted ENNP2‐1 chimera (mENPP2‐1‐T) 48, 49. Of note, HEK293S GnT1^−^ were used to express mENPP2‐1‐T, as described in Kato *et al*. 48, 49. To produce mENPP2‐1‐8xHis (ENPP1 – OPPF 17442), the full‐length gene for ENPP2‐1 containing the native signal sequence (bp 1–2619) was amplified by PCR using Phusion Flash polymerase (Life Technologies, Thermo Fisher Scientific, Hemel Hempstead, UK) and the following primers containing the extensions necessary for ligation‐independent cloning (Forward primer:aggagatataccatgATGGCAAGACAAGGCTGTTTCGG and reverse primer:gtgatggtgatgtttGTCTTCTTGGCTGAAGATTGGCAAATGT). The PCR product was cloned into pOPINEneo using the InFusion method of ligation‐independent cloning as previously reported 59. The vector contains a C‐terminal His8 tag for purification. To purify the mENNP2‐1‐8xHis, 30 mL of Expi293^™^ cells were transfected using the Expi293^™^ Expression System Kit (Invitrogen catalogue no. A14635, Thermo Fisher Scientific). Prior to the day of transfection, Expi293^™^ cells were seeded at 1.5 × 10^6^ cells·mL^−1^ and shaken at 37 °C, 8% CO_2_ in air at 125 r.p.m. for 24 h. For transfection, 30 μg of plasmid DNA (PureLink^®^ HiPure Plasmid Megaprep Kit catalogue no. K2100‐08, Thermo Fisher Scientific) with a A260 : A280 ratio of at least 1 : 1.90, was diluted with 1.5 mL Opti‐MEM^®^ I Reduced Serum Medium (catalogue no. 31985‐070) in a sterile tube. In a separate tube, 80 μL ExpiFectamine^™^ 293 transfection reagent was diluted with 1.5 mL Opti‐MEM^®^ media and both tubes were incubated at room temperature for 5 min. The diluted DNA was then mixed with the transfection reagent and incubated for 20 min at room temperature before adding to 27 mL of cultured Expi293^™^ cells. After 18–20 h, 150 μL of ExpiFectamine^™^ transfection Enhancer 1 and 1.5 mL of ExpiFectamine Transfection^™^ Enhancer 2 were added to the transfected cells. The supernatant containing the mENPP2‐1‐8xHis protein was harvested after 96 h. Secreted protein was purified by automated immobilized metal affinity chromatography followed by gel filtration chromatography using the method of Nettleship *et al*. 60, 61. Briefly, 200 mL of sample was loaded onto a 5 mL HisTrap FF column (GE Healthcare) before washing with 50 mL of 50 mm Tris, pH 7.5, 500 mm NaCl, 30 mm imidazole. This was then repeated until all the samples were loaded. Elution from the HisTrap FF column was of 50 mm Tris, pH 7.5, 500 mm NaCl, 500 mm imidazole and the eluted sample was injected directly onto a HiLoad 16/600 Superdex 200 column. Size exclusion chromatography was performed using 20 mm Tris pH 7.5, 200 mm NaCl and the fractions analysed by SDS/PAGE. The fractions containing the ENPP1 protein were concentrated to 2.1 mg·mL^−1^, based on an extinction coefficient of 1Au = 1 mg·mL^−1^ (0.63 mg final yield) before use. pASK60‐OmpA‐mARTC2.2 6xHis‐Flag tag was a gift from Friedrich Koch‐Nolte (Universitätsklinikum Hamburg‐Eppendorf) and purified as previously described 62.

### Purification of snake venom phosphodiesterase

Phosphodiesterase I (SVP) from *Crotalus adamanteus* venom was purified as previously described 19, 26. Briefly, ~ 2.52 mg dried weight of partially purified SVP (Worthington Biochemical Corporation, Lakewood, NJ, USA) was dissolved in 1 mL of loading buffer (10 mm Tris/HCl pH 7.5, 50 mm NaCl, 10% glycerol) and loaded onto a pre‐equilibrated 1 mL HiTrap blue HP (GE Healthcare). The column was washed with five column volumes (CV) of loading buffer followed by an increasing gradient of KPO_4_ pH 8.0 up to 150 mm. The SVP protein was eluted using 1 m KPO_4_ buffer. Desired fractions were pooled and loaded onto analytical size‐exclusion chromatography Superdex 200 (GE Healthcare) using ÄKTA pure (GE Healthcare) in a buffer composed of 10 mm Tris pH 8.0, 50 mm NaCl, 15 mm MgCl_2_ and 1% glycerol. Concentration of desired fractions (~ 97 kDa molecular weight) was measured using Nanodrop (Thermo Fisher Scientific) and stored at −80 °C.

### Hydrolytic activity assays on ADP‐ribosylated proteins

PARylated and MARylated PARP1 proteins were prepared as described 26 in a reaction buffer containing 50 mm Tris/HCl (pH 8.0), 4 mm MgCl_2_, 50 mm NaCl, 0.2 mm DTT, 200 μm NAD+ (Trevigen) and 130 ng activated DNA (BPS Bioscience, Inc., San Diego, CA, USA). Briefly, for the PAR hydrolysis activity assays, 70 nm PARP1 (PARP1‐HSA; Trevigen) was automodified as described in 26. After 20‐min incubation, PARP1 was passed three times through SpinTrap G‐25 (GE Healthcare). PARylated PARP1 substrate was used in a 10‐μL reaction. For the MARylated PARP1, 1 μm of PARP1‐E988Q was used as a substrate. Reactions were stopped by the addition of PARP inhibitor Olaparib (1 μm). The MgCl_2_ (Sigma) concentration was adjusted to 15 mm to allow full hydrolase activity. Automodified PARP1 was then incubated for indicated times at 30 °C with hydrolytic enzymes in 10‐μL reaction. Concentrations of hydrolytic enzymes used are as indicated in figures. Reactions were stopped by addition of Laemmli loading buffer, samples boiled at 90 °C for 1.5 min and analysed by NuPAGE Novex Bis‐Tris 4–12% gel using MOPS buffer (Invitrogen). Radiolabelled experiments were visualized by autoradiography.

### Thin layer chromatography

The TLC was performed as previously described 26. PARylated PARP1 and MARylated PARP1‐E988Q/GST‐PARP10cd proteins were automodified in the presence of [^32^P]‐labelled NAD^+^ as described above. The product of this reaction was then cleaned up by G25 desalting columns, MgCl_2_ was added to a final concentration of 15 mm and 10‐μL reaction samples were processed by NUDT16, ENPP1, PARG and SVP, as described above. About 1 μL of reaction was spotted onto polyethyleneimine (PEI)‐cellulose plates (Macherey‐Nagel, Polygram CEL 300 PEI/UV254) and developed in 0.15 M LiCl and 0.15 M formic acid. Dried plates were exposed on X‐ray film or visualized by UV254 shadowing.

### Immunoblotting

Fractionated proteins on gradient gels were transferred onto nitrocellulose membranes using Trans‐Blot Turbo Transfer System (Biorad) at 1.3 A/25 V for 20 min. Membranes were blocked in 5% nonfat dry milk (NFDM; Bio‐Rad Laboratories Ltd., Hemel Hempstead, UK) diluted in 0.1% Tween 20‐PBS and subsequently incubated with rabbit polyclonal anti‐PAR (1 : 2000; Trevigen) and mouse monoclonal anti‐6xHis (1 : 4000; Clontech). Primary antibody incubation was then followed by incubation with secondary antibody as indicated and developed with ECL western blotting detection reagent (GE Healthcare).

### Deglycosylation assay

PNGase F and Endo H enzymes were purchased from New England BioLabs and reactions were performed according to the manufacturer's protocols under denaturant conditions using 1 μg of recombinant substrate.

### *In vitro* cell extract modification

K562 NK cell lines (ATCC) were cultured in RPMI‐1640 (+L‐Glutamine) supplemented with 10% inactivated foetal bovine serum (Life Technology, Thermo Fisher Scientific) and 1% penicillin/streptomycin. About 8 × 10^6^ cells were washed twice in PBS and then lysed 20 min in 50 mm Tris/HCl pH 7.5, 150 mm NaCl, 0.5% Triton X‐100, 0.2 mm DTT, 1 μm Olaparib, 4 mm Pefabloc^®^ SC PLUS (Sigma‐Aldrich) at 4 °C. After centrifugation at 15 900 ***g*** for 20 min, proteins in cell extract were quantified using Bradford solution (Biorad) and 100 μg·μL^−1^ BSA standard diluted to 1 μg·μL^−1^ into lysis buffer. Lysate was diluted five times with no‐Triton X‐100 buffer up to 0.6 μg·μL^−1^ protein concentrations. Of diluted lysate, 1 mL was supplemented with 1 μCi (37 kBq) of [^32^P]‐labelled NAD^+^ and 15 mm MgCl_2_. Exactly, 67‐μL extract aliquots were then incubated or not with 1 μm recombinant mARTC2.2 for 15 min at 30 °C. After 15‐min incubation, lysates were additionally incubated or not with several concentrations of NUDT16 and mENPP2‐1‐T for 45 min at 30 °C. Loading sample buffer was added, samples boiled for 4 min at 90 °C and 30 μL was fractionated on SDS/PAGE.

### Preparation of pR‐tagged PARP1 for analysis by LC‐MS/MS

The 6xHis‐*hs*PARP1 (wild‐type) was expressed and purified from *E. coli*, attached to MagneHis beads (Promega Corporation, Madison, WI, USA), and PARylated as described previously 19 with the following changes: PARP1 (final concentration 1 μm) was autoPARylated in the presence of 1 mm β‐NAD^+^ for 30 min at 37 °C. About 60 pmoles of PARylated 6xHis‐*hs*PARP1 was then exposed to 120 pmoles of SVP, 120 pmoles of ENPPs or 600 pmoles of ENPPs for 2 h at 37 °C in the presence of 50 mm Tris pH 7, 150 mm NaCl, 15 mm MgCl_2_ and 1 mm 3‐aminobenzamide. The 6xHis‐hsPARP1 was denatured in 8 m urea, 50 mm Tris pH 7.0 for 10 min at 37 °C and then reduced in 1 mm TCEP (Tris‐(2‐carboxyethyl)phosphine) for 10 min at 37 °C and alkylated in 2 mm CAM (2‐chloroacetamide) for 10 min at 37 °C in the dark. Samples were diluted to final concentrations: 1 m urea, 0.2 m Tris/HCl pH 7.0, 50 mm NaCl, 15 mm MgCl_2_ and 1 mm CaCl_2_. Trypsin (Promega) and LysC (Wako, Richmond, VA, USA) were added at a 1 : 50 enzyme:substrate ratio and digestion was carried out overnight (16–18 h).

### Preparation of pR‐tagged PARP‐10 for analysis by LC‐MS/MS

The catalytic domain of human PARP‐10 (residues 818–1025) was cloned from peGFP‐PARP‐10 into a pBAT4‐derived vector with an N‐terminal 6xHis‐SUMO tag. The construct was transformed and expressed in DE3 Rosetta *E. coli* cells that were cultured to an OD of 0.5 at 37 °C, induced with 0.3 mm IPTG and grown overnight at 16 °C. The cells were harvested the next morning, lysed by sonication in binding buffer (25 mm HEPES pH 7.0, 500 mm NaCl, 20 mm imidazole pH 7.4, 10% glycerol, 20 mm beta‐mercaptoethanol (BME) and SigmaFast Protease Inhibitor (Sigma) at a 1× concentration) and cleared by centrifugation. The supernatant was applied to a 5 mL HisTrap Crude FF column (GE), washed with 10 column volumes of binding buffer and eluted with binding buffer supplemented with imidazole to 250 mm. The eluent was desalted into binding buffer and incubated with 6xHis‐SENP SUMO protease for 2 h at 4 °C at a 1 : 50 enzyme:substrate ratio. Untagged PARP‐10^818–1025^ was then purified further by reverse IMAC on a 1 mL HisTrap Crude FF column (GE) and gel filtration chromatography on a Superose 12 10/300 column (GE) into storage buffer (20 mm Tris pH 7.0, 200 mm NaCl, 5% glycerol and 1 mm DTT). Aliquots were snap frozen in liquid nitrogen and stored at −80 °C. For each reaction, 20 μg of PARP‐10^818–1025^ was incubated with 1 mm NAD^+^ in automodification buffer (20 mm Tris pH 7.5, 50 mm NaCl, 5 mm MgCl_2_ and 20 mm BME) at 30 °C for 30 min to induce mono(ADP‐ribosyl)ation. The reaction was then supplemented with MgCl_2_ to a 15 mm concentration and 20 μg of ENPP‐2‐1‐T was then added to the reaction and incubated at 37 °C for 120 min. The 2× denaturing buffer (200 mm Tris pH 7.5, 3 m guanidine hydrochloride, 2 mm CaCl_2_, 10 mm TCEP and 20 mm CAM) was added to a 1× concentration and incubated at 95 °C in the dark for 10 min. Trypsin and LysC were added at a 1 : 20 enzyme:substrate ratio and incubated at 37 °C overnight. Phosphoribosylated and phosphorylated peptides were then enriched and desalted on in‐house C18 StageTips overlaid with PHOS‐Select IMAC resin (Sigma) and analysed by LC‐MS/MS as previously described 19. Raw data were analysed as previously described 19.

## Conflicts of interest

The authors declare no competing financial interests.

## Author contributions

LP made the initial observation; LP, IA, CMD and AKLL conceived and planned the experiments; LP, CMD, RLM and SEO performed MS/MS experiments; LP, IA, CMD and AKLL wrote the manuscript; LP, CMD, RLM, JEN, NR, KK and ON cloned and purified proteins; all authors read and approved the final version of this manuscript.
